# Supplementary figures and images for: Optimization of cataract surgery follow-up: A standard set of questions can predict unexpected management changes at postoperative week one
Source: PLoS One. 2019 Sep 19;14(9):e0221243. doi: 10.1371/journal.pone.0221243 (PMC6752806; doi:10.1371/journal.pone.0221243)

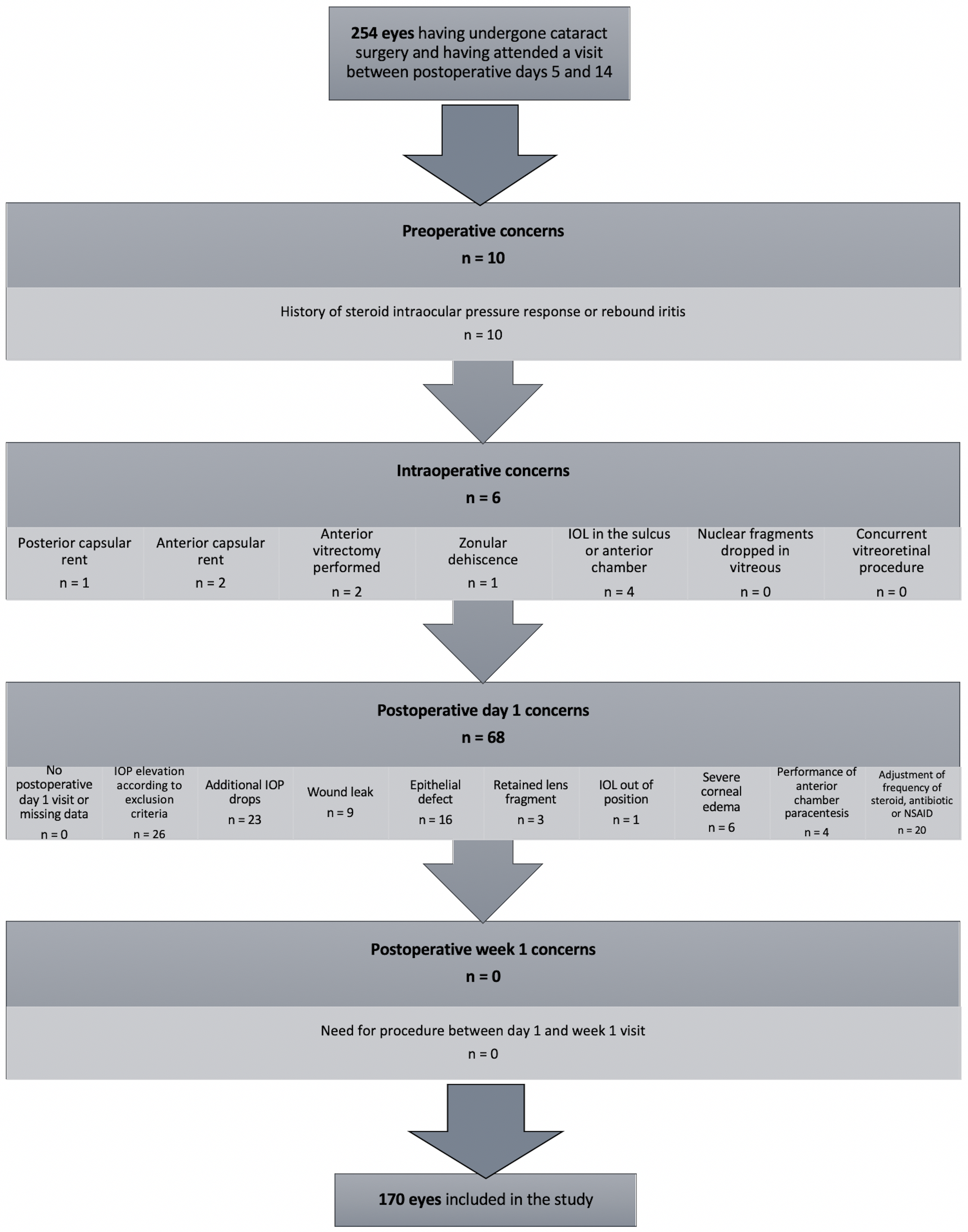

Supplement: S1 Fig — For each timepoint, cases are listed under each subcategory for which an exclusion criterion was met (i.e. some cases may be listed under multiple exclusion criteria). Abbreviations: IOL, intraocular lens; IOP, intraocular pressure; NSAID, nonsteroidal anti-inflammatory drug. (PNG) [file pone.0221243.s001.png]

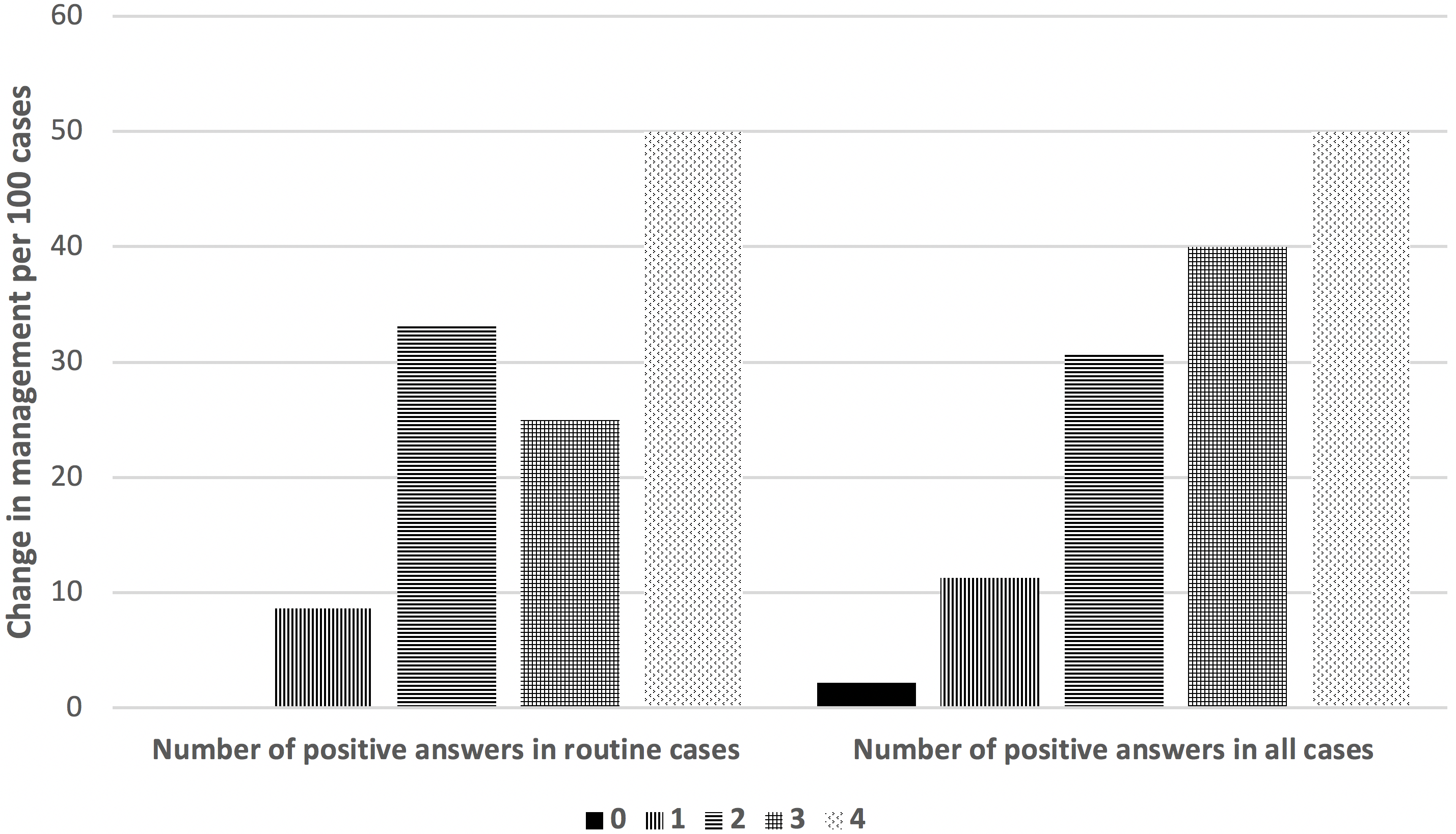

Supplement: S2 Fig — (TIFF) [file pone.0221243.s002.tiff]
