# Supplementary material for: Optimization of cataract surgery follow-up: A standard set of questions can predict unexpected management changes at postoperative week one
Source: PLoS One. 2019 Sep 19;14(9):e0221243. doi: 10.1371/journal.pone.0221243 (PMC6752806; doi:10.1371/journal.pone.0221243)
Supplement: S1 Table — (DOCX) [file pone.0221243.s003.docx]

**S1 Table.** Incidence of unexpected management changes at postoperative week 1.*^§^*

|  | No. (%) |
| --- | --- |
| Change in drop regimen taper | 6 (3.5) |
| - Change in antibiotic regimen - Change in steroid regimen - Change in NSAID regimen | 1 (0.6)  1 (0.6)  5 (2.9) |
| Additional eye drops | 2 (1.2) |
| - IOP-lowering eye drops - Non-IOP-lowering eye drops | 0 (0.0)  2 (1.2) |
| Procedure performed (excluding suture removal) | 0 (0.0) |
| Referral to ophthalmology specialist | 2 (1.2) |
|  | No. (%) |
| Change in drop regimen taper  Change in antibiotic regimen  Change in steroid regimen  Change in NSAID regimen | 6 (3.5)  1 (0.6)  1 (0.6)  5 (2.9) |
| Additional eye drops  IOP-lowering eye drops  Non-IOP-lowering eye drops | 2 (1.2)  0 (0.0)  2 (1.2) |
| Procedure performed (excluding suture removal) | 0 (0.0) |
| Referral to ophthalmology specialist | 2 (1.2) |

*Abbreviations: IOP intraocular pressure; NSAID nonsteroidal anti-inflammatory drug*

*^§^Some cases had more than one management changes*
