# Supplementary material for: Optimization of cataract surgery follow-up: A standard set of questions can predict unexpected management changes at postoperative week one
Source: PLoS One. 2019 Sep 19;14(9):e0221243. doi: 10.1371/journal.pone.0221243 (PMC6752806; doi:10.1371/journal.pone.0221243)
Supplement: S2 Table — (DOCX) [file pone.0221243.s004.docx]

| Table/Fig. # | Mean | SD | Number | Percentage | Statistical method | P value | # of cases |
| --- | --- | --- | --- | --- | --- | --- | --- |
| **Table 2** |  |  |  |  |  |  |  |
| Age, years | 69.47 | 10.37 |  |  | Descriptive statistics |  | 170 |
| Gender: Male |  |  | 78 | 45.9 | Descriptive statistics |  | 78 |
| Gender: Female |  |  | 92 | 54.1 |  |  | 92 |
| Race/Ethnicity: White |  |  | 103 | 60.6 | Descriptive statistics |  | 103 |
| Race/Ethnicity: Black/African American |  |  | 25 | 14.7 |  |  | 25 |
| Race/Ethnicity: Asian |  |  | 7 | 4.1 |  |  | 7 |
| Race/Ethnicity: Hispanic/Latino |  |  | 10 | 5.9 |  |  | 10 |
| Race/Ethnicity: Other |  |  | 10 | 5.9 |  |  | 10 |
| Race/Ethnicity: Not available/Declined to declare |  |  | 10 | 5.9 |  |  | 10 |
| Operative eye: OD |  |  | 84 | 49.4 | Descriptive statistics |  | 84 |
| Operative eye: OS |  |  | 86 | 50.6 |  |  | 86 |
| Second eye: Yes |  |  | 65 | 38.2 | Descriptive statistics |  | 65 |
| Second eye: No |  |  | 105 | 61.8 |  |  | 105 |
| **Table 3** |  |  |  |  |  |  |  |
| Pain: Yes |  |  | 2 | 40.0 | Fisher exact test | .018 | 5 |
| Pain: No |  |  | 6 | 3.6 |  |  | 165 |
| Redness: Yes |  |  | 3 | 42.9 | Fisher exact test | .002 | 7 |
| Redness: No |  |  | 5 | 3.1 |  |  | 163 |
| Unhappy with vision: Yes |  |  | 5 | 27.8 | Fisher exact test | <.0001 | 18 |
| Unhappy with vision: No |  |  | 3 | 2.0 |  |  | 150 |
| Decrease in vision: Yes |  |  | 4 | 40.0 | Fisher exact test | <.0001 | 10 |
| Decrease in vision: No |  |  | 3 | 1.9 |  |  | 159 |
| Floaters: Yes |  |  | 0 | 0.0 | Fisher exact test | >.99 | 17 |
| Floaters: No |  |  | 7 | 4.6 |  |  | 152 |
| Flashes: Yes |  |  | 0 | 0.0 | Fisher exact test | >.99 | 9 |
| Flashes: No |  |  | 8 | 5.0 |  |  | 161 |
| Understanding of drops: Yes |  |  | 5 | 3.1 | Fisher exact test | .005 | 160 |
| Understanding of drops: No |  |  | 3 | 33.3 |  |  | 9 |
| 7-question set: ≥1 positive answers |  |  | 8 | 16.0 | Fisher exact test | <.0001 | 50 |
| 7-question set: No positive answers |  |  | 0 | 0.0 |  |  | 120 |
| **Table S1** |  |  |  |  |  |  |  |
| Change in drop |  |  | 6 | 3.5 | Descriptive statistics |  | 6 |
| Change in antibiotic |  |  | 1 | 0.6 |  |  | 1 |
| Change in steroid |  |  | 1 | 0.6 |  |  | 1 |
| Change in NSAID |  |  | 5 | 2.9 |  |  | 5 |
| Additional drops |  |  | 2 | 1.2 | Descriptive statistics |  | 2 |
| Additional IOP-lowering drops |  |  | 0 | 0.0 |  |  | 0 |
| Additional non-IOP-lowering drops |  |  | 2 | 1.2 |  |  | 2 |
| Procedure performed (excluding suture removal) |  |  | 0 | 0.0 | Descriptive statistics |  | 0 |
| Referral to specialist |  |  | 2 | 1.2 | Descriptive statistics |  | 2 |
| **Fig. 1** |  |  |  |  |  |  |  |
| Routine cases |  |  |  |  |  |  |  |
| Number of positive answers: 0 |  |  | 0 | 0.0 | Descriptive statistics |  |  |
| Number of positive answers: 1 |  |  | 3 | 8.6 |  |  | 35 |
| Number of positive answers: 2 |  |  | 3 | 33.3 |  |  | 9 |
| Number of positive answers: 3 |  |  | 1 | 25.0 |  |  | 4 |
| Number of positive answers: 4 |  |  | 1 | 50.0 |  |  | 2 |
| All cases |  |  |  |  |  |  |  |
| Number of positive answers: 0 |  |  | 4 | 2.2 | Descriptive statistics |  | 181 |
| Number of positive answers: 1 |  |  | 6 | 11.3 |  |  | 53 |
| Number of positive answers: 2 |  |  | 4 | 30.8 |  |  | 13 |
| Number of positive answers: 3 |  |  | 2 | 40.0 |  |  | 5 |
| Number of positive answers: 4 |  |  | 1 | 50.0 |  |  | 2 |

**S2 Table.** Data and methods used to reach the conclusions drawn in the manuscript.
